# Rapid brain MRI protocols reduce head computerized tomography use in the pediatric emergency department

**DOI:** 10.1186/s12887-020-1919-3

**Published:** 2020-01-13

**Authors:** Sriram Ramgopal, Sabrina A. Karim, Subramanian Subramanian, Andre D. Furtado, Jennifer R. Marin

**Affiliations:** 1 Division of Emergency Medicine, Ann and Robert H. Lurie Children’s Hospital of Chicago, Feinberg School of Medicine, 225 E Chicago Ave, Box 62, Chicago, IL 60611 USA; 2 University of Pittsburgh School of Medicine, Pittsburgh, PA USA; 3 Division of Pediatric Radiology, Children’s Hospital of Pittsburgh, Department of Radiology, University of Pittsburgh School of Medicine, Pittsburgh, PA USA; 4 Department of Emergency Medicine, University of Pittsburgh School of Medicine, Pittsburgh, PA USA

**Keywords:** Fast MRI, Quickbrain MRI, Rapid MRI, Emergency medicine, Neuroimaging

## Abstract

**Background:**

Rapid magnetic resonance imaging (MRI) protocols may be effective in the emergency department (ED) to evaluate nontraumatic neurologic complaints. We evaluate neuroimaging (rapid MRI [rMRI]), head computerized tomography [HCT], and full MRI) use following widespread implementation of rMRI protocols in a pediatric emergency department (ED).

**Methods:**

We conducted a retrospective study in a tertiary care pediatric ED of encounters with neuroimaging during two 9-month periods: one prior to (control period) and one after generalized availability of 4 rMRI protocols (rMRI period). The primary outcome was differences in neuroimaging rates between the two periods. Secondary outcomes included ED process measures, unsuccessful imaging, and undetected pathology, with full MRI within 14 days as the reference standard.

**Results:**

There were 1052 encounters with neuroimaging during the control and 1308 during the rMRI periods. Differences in neuroimaging between periods were 27.7% for rMRI (95% CI, 24.4, 31.0), − 21.5% for HCT (95% CI, − 25.5, − 17.5), and − 6.2% for full MRI (95% CI, − 9.3, − 3.1%.) Time to imaging (182 [IQR 138–255] versus 86 [IQR 52–137] minutes) as well as ED length of stay (396 [IQR 304–484] versus 257 [IQR 196–334] minutes) was longer for rMRI versus HCT (*p* < 0.01). Between the control and rMRI periods, there were differences in types of neuroimaging performed for patients with altered mental status, headache, seizure, shunt dysfunction, stroke, syncope, trauma, vomiting, infection, and other neurologic complaints (*p* < 0.05). rMRI studies were unsuccessful in 3.6% of studies versus 0.0% of HCTs (*p* < 0.01). The 22 unsuccessful rMRI studies were unsuccessful due to artifacts from dental hardware (*n* = 2) and patient motion (*n* = 20). None of the rMRI studies with full MRI follow-up imaging had undetected pathology; the false negative rate for the HCT exams was as high as 25%.

**Conclusions:**

After routine ED use of 4 rMRI protocols, there was a more than 20% decrease in HCT use without missed diagnoses. Time to neuroimaging and length of stay were longer for rMRI than HCT, with higher rates of unsuccessful imaging. Despite these limitations, rMRI may be an alternative to HCT for nontraumatic complaints in the ED.

## Background

Brain magnetic resonance imaging (MRI) is an accurate, safe, non-radiating cross-sectional imaging modality. Historically, MRI use in children, particularly patients evaluated in the emergency department (ED), has been limited by the exam duration and need for sedation in many cases. Consequently, head computed tomography (HCT) is often the default imaging modality. HCT carries the benefits of being a rapid, readily available, and generally more inexpensive neuroimaging modality. For example, multicenter data from pediatric hospitals between the years 2009–2013 suggest that 56 patients per 1000 encounters (ED or inpatient) get a CT, with the majority (60%) being HCTs [1]. Additionally, HCT is preferred in cases of trauma within the context of Advanced Trauma Life Support guidelines [2]. Despite these advantages, HCT carries the carcinogenic risks associated with radiation, which are particularly concerning in pediatric patients [3, 4]. Additionally, HCT is limited in its evaluation of posterior fossa lesions as well as findings related to ischemic stroke [5].

Rapid-sequence magnetic resonance imaging of the brain (rMRI) has gained acceptance as an alternative to HCT in children because of the speed of image acquisition. Previous studies highlight experiences with rMRI protocols for a single indication, including ventricular shunt malfunction [6–10], stroke [11], and abusive head trauma [12, 13]. These studies demonstrate a role for rMRI as a radiation-sparing alternative to HCT. In patients evaluated for ventricular shunt malfunction, for example, retrospective studies have suggested that rapid MRI is not inferior to HCT, with comparable measures of diagnostic accuracy [9, 14]. Additionally, HCT imaging is frequently used to also evaluate other common pediatric neurologic complaints in the ED, including headache, syncope, and seizure, for example [15]. rMRI imaging may be an alternative to HCT for these patients as well. At present, there are very limited single center data which suggest that availability of 24/7 MRI facilities may be associated with increased rates of MRI [16]. No investigation to date, however, has identified if availability of rapid MRI protocols may allow for decreased HCT utilization.

The primary objective of this study was to evaluate the rates of neuroimaging (rMRI, HCT, and full MRI) before and after widespread implementation of four rMRI protocols in the ED. Secondary objectives were to evaluate ED process measures within these time periods, specifically, time to neuroimaging, total ED length of stay (LOS), and rates of unsuccessful initial imaging, follow-up imaging, and undetected pathology.

## Methods

### Study setting

We performed a single center retrospective study in a tertiary care freestanding children’s hospital ED with an annual volume of over 80,000 patients. Ours is the only children’s hospital in the region and is part of a large, integrated healthcare delivery system that includes 42 hospitals with a shared electronic medical record, including the radiology Picture Archiving and Communication System. Since November 2017, our ED implemented widespread ED availability of 4 distinct rMRI neuroimaging protocols (Table 1): ventricular shunt evaluation, abusive head trauma screen, stroke, and nonspecific neurologic complaints (e.g. headache, seizure, altered mental status, vomiting). Prior to this time, although some protocols were available, they were not yet utilized as part of routine ED practice. rMRI are performed using five MRI scanners: 1) three GE Signa 1.5 T, 2) one GE Signa 3 T (GE Healthcare, Chicago, IL), and 3) one Siemens Skyra 3 T (Siemens Medical Solutions, Malvern, PA). The protocols performed are the same for all patients irrespective of age.

**Table 1:** rMRI protocols

Protocol	Sequences	Duration
Shunt	Coronal T2 SSFSE	7 min
	Sagittal T2 SSFSE	
	Axial T2 SSFSE	
	Axial 3D SWAN	
Abusive head trauma screen	Axial DWI	16 min
	Axial GRE	
	Axial T2 Propeller	
	Coronal T1 FLAIR	
	Axial 3d SWAN	
	Axial fast FLAIR	
	Axial T2 SSFSE	
Stroke	Axial DWI	22 min
	3D ASL	
	Axial T2 FLAIR	
	Axial 3D SWAN	
	3D Time-of-flight 3-slab MRA	
Neurologic	DWI	7 min
	Axial GRE	
	Axial T2 SSFSE	
	Axial T2 FLAIR	
	Sagittal T1 FSPGR	

*SSFSE* single-shot fast spin-echo sequence, *SWAN* Susceptibility-weighted angiography, *DWI* Diffusion-weighted magnetic resonance imaging, *GRE* gradient echo, *FLAIR* Fluid-attenuated inversion recovery, *ASL* arterial spin labeling, *MRA* magnetic resonance angiography, *FSPGR* fast spoiled gradient echo. Protocol durations include the localizer time

Neuroimaging studies performed as part of an ED encounter at our institution are not done with sedation; and, if sedation is needed, imaging is performed once the patient is admitted (or scheduled as an outpatient.) It is possible, however, for patients to receive oral, intranasal, or intravenous anxiolysis (e.g. midazolam, dexmedetomidine) to facilitate neuroimaging. We did not specifically identify use of sedative agents utilized for the purposes of neuroimaging in the context of this study.

### Study cohort

We included neuroimaging studies (rMRI brain, HCT, and full MRI) performed as part of an ED encounter during two periods: November 1, 2016 to July 31, 2017 (control period) and November 1, 2017 to July 31, 2018 (rMRI period). We excluded studies performed for patients > 18 years-old. HCT remains the modality of choice for acute head trauma [2], therefore, we excluded imaging studies in patients > 12 months of age if the imaging was for trauma as documented as part of the “indication” section of the radiology report. We included studies in patients with possible trauma ≤12 months of age because of a dedicated rMRI protocol to evaluate for abusive head trauma in this age group, which may or may not be associated with a corroborating history or physical examination for an acute traumatic injury.

### Neuroimaging studies

For each encounter, we defined the “index” neuroimaging study as the first study (non-contrast rMRI, non-contrast HCT, or full MRI with and/or without contrast) performed in the ED. We included only one ED encounter per patient per study period, and, a priori, determined that if a patient had multiple encounters during one of the two study periods, only the last encounter for that period would be included.

We assessed for follow-up imaging after the index study, defined as any neuroimaging (including outpatient, inpatient, and ED) performed within 14 days of the index study. For cases when more than one study was performed within this period, the earliest was considered the follow-up study. We further evaluated within this 14-day period if a full MRI was performed, even if it was not the first follow-up imaging study performed.

For index and follow-up neuroimaging studies, two pediatric emergency medicine physicians (S.R. and J.R.M), blinded to the clinical history and examination and to the type of imaging performed, reviewed all attending radiology interpretations and classified them into one of four categories: a) *positive*, in which the findings from neuroimaging would require further testing, admission, or subspecialist consultation; b) *negative*, defined as no acute pathology identified or would typically not require further investigation or follow up, c) *unknown*, in which the results could not be classified as positive or negative, and d) *unsuccessful*, defined as a study which was sufficiently limited to preclude radiology interpretation. For this process, the investigators both reviewed approximately 50% of all of the included imaging studies in order to determine if there was sufficient agreement, defined, a priori*,* as Kappa ≥0.70 [17], before reviewing the remainder independently. For discordances between the two raters as well as interpretations deemed *unknown,* an attending radiologist (also blinded as described above) reviewed the interpretation and classified the study as *positive* or *negative*.

For patients with a follow-up full MRI within 14 days of the index study and for whom the index scan was either a HCT or rMRI during the rMRI study period, two attending radiologists, blinded to the index study modality, independently reviewed the full MRI interpretations and compared them to HCT or rMRI index exam interpretations to evaluate for undetected pathology in these studies. Specifically, they categorized index studies as: true positive (including possible progression of disease), true negative, false positive, and false negative.

### Data acquisition

We used Centricity RIS-IC (version 6.0; GE Healthcare, Chicago, IL) to determine which patients had neuroimaging during the two study periods. These data were then linked to radiology results obtained using mPower Clinical Analytics (version 3.2.1; Burlington, Massachusetts) using unique accession numbers. Patient-related data were obtained from data harbored by the electronic health record using the business intelligence platform SAP BusinessObjects (SAP, Waldorf, Germany). We extracted the following for each encounter: patient age, sex, race, ED chief complaint, reason for neuroimaging, emergency severity index (ESI) score, time of arrival, time of final disposition, ED disposition (admitted, discharged, transferred to another institution, or deceased), time and duration of index neuroimaging, and follow-up neuroimaging. The ESI is assigned by a triage nurse and is an ED triage algorithm that stratifies patients into 5 groups on the basis of acuity and resource needs, with 1 representing the most acute [18]. ED chief complaint is also assigned by a triage nurse at the time of arrival and is based on a standardized list of 109 complaints.

### Outcomes

The primary outcome was rates of neuroimaging (rMRI, HCT, and full MRI) between both periods. Secondary outcomes were time to index neuroimaging study, ED LOS, rates of unsuccessful index imaging, follow-up imaging, and undetected pathology on initial imaging for patients with rMRI or HCT as the index study and for whom a subsequent MRI was performed within 14 days of initial evaluation.

We also assessed imaging patterns across the entire ED population (not exclusively those with neuroimaging) and evaluated use of any neuroimaging as well as modality-specific rates for each time period. We applied the same exclusion criteria as in our primary cohort and identified those with trauma using the International Classification of Diseases, revision-10 codes, S00-T88.

### Data analysis

We summarized demographics between the two time periods using proportions. We calculated interrater reliability using Cohen’s Kappa statistic [17]. All continuous data were nonparametric. For our primary outcome, we compared the difference in proportions for each type of neuroimaging (HCT, rMRI, and full MRI) between the two periods and analyzed changes in neuroimaging by chief complaint and assessed for differences using chi-squared tests. For secondary outcomes we compared results using chi-squared and Wilcoxon rank-sum tests. For the evaluation of undetected pathology on index imaging during the rMRI period, we calculated the false negative rate of rMRI or HCT using full MRI performed within 14 days as the reference standard. We further assessed the accuracy of these index studies by calculating the sensitivity, specificity, positive predictive value, negative predictive value, positive likelihood ratio and negative likelihood ratio with 95% confidence intervals. Analyses were performed using the epiR (version 0.9.99) package for R, version 3.5.1 (R Foundation for Statistical Computing, Vienna, Austria). This study was approved with a waiver of informed consent by the University of Pittsburgh Human Research Protection Office.

### Exploratory analysis

In order to assess the effect of our assumptions regarding non-accidental trauma (i.e. including patients < 12 months with any trauma) on the primary outcome, we performed an additional analysis excluding all patients with trauma regardless of age. Additionally, we reported the number of rMRI performed and the percent of neuroimaging performed as rMRI during each week of the rMRI period.

## Results

### Demographics

**Fig. 1:**
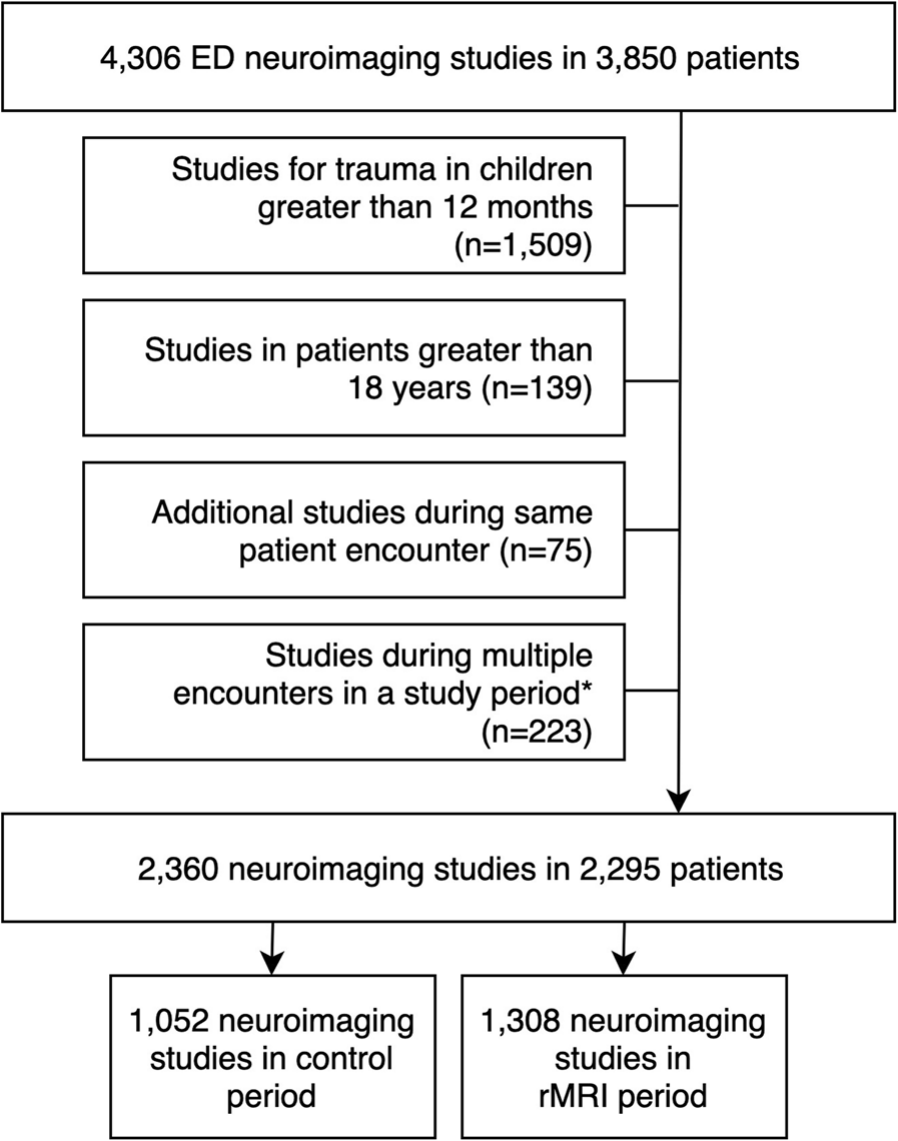
Initial (“index”) neuroimaging studies in the cohort. *For patients with > 1 encounter during a study period, imaging during the latest encounter was retained and remainder excluded. These 223 excluded studies originated from 136 patients

A total of 4306 neuroimaging studies were performed during the two periods. After applying exclusions, there were 2360 index studies (1052 in the control period; 1308 in the rMRI period) among 2295 patients (Fig. 1). There was a higher proportion of high-acuity patients (ESI 1 and 2) as well as of patients admitted to the intensive care unit in the control period compared to the rMRI period (Table 2).

**Table 2:** Demographics of patients who received ED neuroimaging

Variable	All encounters with neuroimaging (*N* = 2360) n (%)	Control period (*N* = 1052) n (%)	rMRI period (*N* = 1308) n (%)
Age			
< 1 year	734 (31.1)	327 (31.1)	407 (31.1)
1 to < 4 years	283 (12.0)	134 (12.7)	149 (11.4)
4 to < 12 years	621 (26.3)	278 (26.4)	343 (26.2)
12 to < 19 years	722 (30.6)	313 (29.8)	409 (31.3)
Number male	1257 (53.3)	660 (50.5)	597 (56.7)
Race			
White	1769 (75.0)	778 (74.0)	991 (75.8)
Black	458 (19.4)	198 (18.8)	260 (19.9)
Other	61 (2.6)	30 (2.9)	31 (2.4)
Unknown	72 (3.1)	46 (4.4)	26 (2.0)
Weekend presentation	566 (24.0)	247 (23.5)	319 (24.4)
Day time (06:00–17:59) presentation	1344 (56.9)	619 (58.8)	725 (55.4)
ESI Codes			
4 or 5	204 (8.6)	62 (5.9)	142 (10.9)
3	1.769 (75.0)	755 (71.8)	1014 (77.5)
2	340 (14.4)	208 (19.8)	132 (10.1)
1	35 (1.5)	19 (1.8)	16 (1.2)
Not listed	12 (0.5)	8 (0.8)	4 (0.3)
ED Disposition			
Discharged	1219 (54.6)	538 (51.1)	751 (57.4)
PICU	367 (15.6)	194 (18.4)	173 (13.2)
Admitted	693 (29.4)	316 (30.0)	377 (28.8)
Transferred	10 (0.4)	4 (0.4)	6 (0.5)
Expired	1 (0.0)	0 (0.0)	1 (0.1)

*rMRI* rapid magnetic resonance imaging, *ESI* Emergency Severity Index, *ED* emergency department, *PICU* pediatric intensive care unit

### Primary outcome

Use of rMRI as the index ED imaging modality was 10.8% during the control period and 38.5% in the rMRI period (percent difference 27.7%; 95% CI 24.4, 31.0%); HCT use in the control and rMRI periods were 70.0 and 48.5%, respectively (percent difference, − 21.5%; 95% CI, − 25.5, − 17.5%) (Table 3). These differences all reached statistical significance (*p* < 0.01).

**Table 3:** Neuroimaging by modality between time periods and secondary outcomes comparing time periods

Variable	Control period (*N* = 1052) n (%)	rMRI period (*N* = 1308) n (%)	Difference in percent (95% CI)	*P*
rMRI	114 (10.8)	504 (38.5)	27.7 (24.4, 31.0)	< 0.01
Head CT	736 (70.0)	634 (48.5)	−21.5 (−25.5, −17.5)	< 0.01
Full MRI	202 (19.2)	170 (13.0)	−6.2 (−9.3, −3.1)	< 0.01
Time to neuroimaging in minutes; median (IQR)	119 (64–193)	139 (84–208)	–	< 0.01
Total ED LOS in minutes; median (IQR)	304 (231–387)	304 (232–397)	–	0.82
Any follow-up neuroimaging within 14 days, n (%)	130 (12.4)	169 (12.9)	0.6 (−2.2, 3.3)	0.72
Full MRI within 14 days, n (%)	91 (8.7)	130 (10.0)	1.3 (−1.1, 3.7)	0.36
Time to follow up neuroimaging, n (%)				0.96
1–2 days	100 (76.9)	128 (75.7)	−1.2 (−11.6, 9.2)	
3–7 days	16 (12.3)	21 (12.4)	0.1 (−7.5, 7.8)	
8–14 days	14 (10.8)	20 (11.8)	1.1 (−6.8, 9.0)	

*rMRI* rapid MRI, *CT* computerized tomography, *MRI* magnetic resonance imaging, *IQR* interquartile range, *ED* emergency department, *LOS* length of stay

### Neuroimaging by chief complaint

Neuroimaging studies were performed in patients with 15 different complaints across the two time periods. Compared to the control period, during the rMRI period, there were statistically significant differences in neuroimaging patterns for altered mental status, headache, seizure, shunt dysfunction, stroke, syncope, trauma, vomiting, infection, other neurologic complaints, and other complaints (Table 4). Relative rates of neuroimaging were not statistically significantly different in patients with abdominal complaints, eye complaints, fussiness, nonaccidental trauma, and vomiting with diarrhea, respiratory complaints, and brief resolved unexplained events. During the control period, there were 114 (10.8%) rMRI studies, including 85 (74.6%) abusive head trauma screen protocols, 14 (12.3%) shunt protocols, 7 (6.1%) stroke protocols, and 8 (7.0%) neurologic protocols. During the rMRI period, 504/1308 (38.5%) of studies were rMRI protocols including 330 (65.5%) neurologic protocols, 82 (16.3%) abusive head trauma screen protocols, 46 (9.1%) stroke protocols, and 46 (9.1%) shunt protocols.

**Table 4:** Neuroimaging performed in the control and rMRI periods for those complaints with significant differences between the time periods. For each chart, the y axis represents the percent of studies in each time period

Category	Control Period Number of rMRI/total number in category (%)	rMRI period Number of rMRI/total number in category (%)	*P*
Headache	13/217 (6.0)	135/284 (47.5)	< 0.01
Trauma in infant	17/196 (8.7)	44/253 (17.4)	0.02
Seizure	13/135 (9.6)	73/200 (36.5)	< 0.01
Shunt evaluation	2/90 (2.2)	30/70 (42.9)	< 0.01
Neurologic complaints	4/65 (6.2)	29/71 (40.8)	< 0.01
Altered mental status	5/57 (8.8)	17/54 (31.5)	< 0.01
Vomiting	7/44 (15.9)	31/66 (47.0)	< 0.01
Non-accidental trauma	20/46 (43.5)	22/46 (47.8)	0.29
Infection	5/33 (15.2)	20/40 (50.0)	< 0.01
Syncope	2/30 (6.7)	13/33 (39.4)	< 0.01
Eye complaint	2/25 (8.0)	11/34 (32.4)	0.07
Fussiness	10/17 (58.8)	14/26 (53.8)	0.70
Stroke	1/16 (6.3)	7/14 (50.0)	0.02
Respiratory	0/9 (0.0)	6/15 (40.0)	0.09
Vomiting with diarrhea	2/8 (25.0)	8/15 (53.3)	0.42
BRUE	4/12 (33.3)	7/10 (70.0)	0.07
Abdominal complaint	2/8 (25.0)	8/13 (61.5)	0.16
Other*	5/44 (11.4)	29/64 (45.3)	< 0.01

*Other sickle cell disease with pain (*n* 6), dehydration, neck pain (*n* 5 each), ingestion/overdose (*n* 4), abnormal labs, cardiac arrest, hematemesis/bloody stool (*n* 3 each), congestion, ear pain, eye injury (*n* 2 each) allergic reaction, back pain, constipation, cough, croup, diabetes, genitourinary complaint, hemophilia, poor feeding, rash, sore throat, wound evaluation (*n* 1 each), and otherwise unclassified general medical complaint (*n* 67). BRUE, brief resolved unexplained event

### Time to neuroimaging and unsuccessful neuroimaging

The median time to neuroimaging in the control cohort was significantly shorter than in the rMRI cohort. Total ED LOS in the two periods were similar, as were rates of follow up imaging. There were differences in time to neuroimaging and total ED LOS when comparing imaging modalities during the rMRI time period, with HCT having the shortest times (Table 5). Across both periods, 22/618 (3.6%) rMRI, 0/1370 (0.0%) HCT, and 11/372 (3.0%) full MRI studies were unsuccessful (*p* < 0.01). Reasons for the 22 unsuccessful rMRI studies were artifacts caused by dental hardware (*n* = 2) and patient motion (*n* = 20). Seven abusive head trauma screen rMRI, 12 neurologic, and 3 stroke protocols were unsuccessful. The median age of patients with unsuccessful rMRI was 1.69 years (IQR 0.36–3.70), compared to 5.19 years (IQR 0.38–12.8) for those with successful rMRI (*p* = 0.12).

**Table 5:** Secondary outcomes for patients in the rMRI period, by index study modality

Variable	rMRI (*N* = 504)	Head CT (*N* = 634)	Full MRI (*N* = 170)	*P* value
Time to neuroimaging in minutes; median (IQR)	182 (138–255)	86 (52–137)	200 (146–262)	< 0.01
Total ED LOS in minutes; median (IQR)	396 (304–484)	257 (196–334)	338 (269–420)	< 0.01
Any follow-up neuroimaging within 14 days, n (%)	49 (9.7)	95 (15.0)	25 (14.7)	0.02
Full MRI within 14 days, n (%)	36 (7.1)	82 (12.9)	N/A	< 0.01
Time to follow up neuroimaging, n (%)				0.77
1–2 days	35 (71.4)	75 (78.9)	18 (72.0)	
3–7 days	8 (16.3)	9 (9.5)	4 (16.0)	
8–14 days	6 (12.2)	11 (11.6)	3 (12.0)	

### Follow-up neuroimaging

Index studies were deemed positive for 16.3 and 11.4% of all neuroimaging studies during the control and rMRI periods, respectively (Additional file 2: Table S1). For the classification of imaging results, the two pediatric emergency medicine physicians demonstrated substantial agreement (κ-statistic of 0.71 [95% CI 0.67–0.72]). There were 118 patients who received a full MRI within 14 days of the index study during the rMRI period with 82 (69.5%) having had an index HCT and 36 (30.5%) having had an rMRI. Both radiologists determined the rate of undetected pathology (false negative rate) was 0% for rMRI; for HCT, one radiologist found a false negative rate of 18% and the other of 25%. rMRI was found to be both highly sensitive and specific. HCT demonstrated a specificity of 94–95% and a sensitivity of 75–82% (Additional file 3: Table S2).

### Overall neuroimaging rates

In the analysis comparing rates of neuroimaging among all ED encounters for nontraumatic complaints, there were 33,117 encounters in the control period and 35,582 in the rMRI period. There was an increase in the percent of patients who had any neuroimaging between these periods, with the increase driven by rMRI use (Additional file 4: Table S3).

### Exploratory analyses

When we excluded all patients < 12 months with trauma (*n* = 811), changes in the rates of neuroimaging were similar to those demonstrated in the primary analysis (Additional file 5: Table S4). In light of the increased proportion of high acuity patients in the control group, we evaluated the association between ESI and HCT using multivariable logistic regression and found that in addition to ESI, time period remained independently associated with HCT utilization after consideration of potential confounders (Additional file 6: Table S5). We noted an increasing number and percent of rMRI performed during each week of the rMRI period (Additional file 1: Figure S1).

## Discussion

Following implementation of 4 standardized rMRI protocols in a pediatric ED, we identified an increase in rMRI imaging that was associated with a significant reduction in HCT use. There were no differences in rates of follow-up imaging and in the group of patients for whom a full MRI was performed within 2 weeks of the index study, there were no false negative rMRI studies. In addition to the limitation in radiation exposure from HCT use, these data support potentially limiting subsequent full MRI imaging, particularly given the need for sedation in some cases, and the cost associated with such imaging.

The benefit of rMRI for shunt dysfunction has been previously investigated [7–10], as has the use of rMRI as a screening tool for nonaccidental trauma [12, 13]. Few studies have reported on the generalized use of rMRI for other indications, and none specifically in the ED setting. Missios, et al., reported on an institution-wide registry of 1146 patients who received rMRI. Two patients were found to have previously undetected pathology on subsequent neuroimaging [19]. Another evaluation of 101 rMRI studies reviewed by a radiologist and neurosurgeon suggested that these studies served as adequate screening evaluations and that such imaging had a low requirement (5%) for follow up full MRI [20]. The findings from our study build upon these prior studies, specifically reporting on ED patients, and demonstrating a concomitant decline in HCT use.

Widespread rMRI implementation includes several barriers. We observed a longer time to neuroimaging and LOS for patients undergoing rMRI compared to HCT. Other studies have reported similar findings [9, 14, 21]. Our ED has a dedicated HCT scanner, compared with the MRI machines which are located on a different floor. Equipment location as well as the preparation time that is needed for screening prior to MRI imaging are likely contributors to the longer times associated with rMRI. It is likely that in the future these times will be reduced as the technology becomes less expensive and more easily accessible. Nonetheless, for the majority of patients, who are clinically stable and for whom the longer time to neuroimaging is unlikely to affect clinical outcomes, this additional time is likely outweighed by the patient benefits. A higher proportion of rMRI studies were unsuccessful compared with HCT in our study. While these studies are “rapid,” they remain longer in duration than a non-contrast HCT. The cost of an MRI Is often cited as a barrier to its use over CT [22]. However, according to hospital charge data at our institution, that for a non-contrast brain MRI is comparable to that for a head CT. Although this may not be consistent across all institutions, and more importantly, the insurance reimbursement rate may not be the same, it is likely that the current cost differences are not as discrepant as once thought. The charge for a rMRI if coded appropriately is significantly less than that for the full MRI. Other concerns highlighted in surveys include lack of rMRI access during evenings or weekends [22]. Finally, it is critical to implement appropriate MRI sequences specific to the diagnosis of interest. While we found no missed diagnoses on rMRI, a previous study reporting on a FIESTA-based protocol found a significant false negative rate, including a missed venous sinus thrombosis and subdural hemorrhage [23]. We did observe that a higher proportion of patients received any neuroimaging in the rMRI period compared with the control. This may reflect a lower threshold to obtain imaging given the availability of rMRI during this period. To what extent this increase represents overuse warrants further exploration.

The findings from this study are subject to limitations. As a single center retrospective study, our results do not generalize to all EDs, particularly those outside of children’s hospitals. We attempted to blind the physicians who reviewed study interpretations, but subtle clues may have biased them in their assessment of positivity. By not excluding infants < 12 months with trauma to ensure inclusion of possible abusive head trauma, we invariably included some patients with acute trauma, for whom HCT is the more appropriate study. However, this would have biased the results towards an underestimation of the rMRI rate and overestimation of the HCT rate; and, a sensitivity analysis excluding these patients produced similar results. While the lower acuity in the rMRI period may have contributed to the lower HCT rate during this period, the time period variable was also independently associated with HCT, indicating that the decrease in HCT use would not completely be explained by the differences in ESI across the two periods. Although it is possible that patients received follow-up neuroimaging outside of our health system resulting in an underestimated rate of follow-up imaging, we would expect this number to be small and also similar between the two time periods. For those patients with multiple encounters during a study period, we retained the last visit. It is possible that imaging decisions at this visit were influenced by imaging performed during prior visit(s). However, these excluded studies represent a small fraction (223 patients from 136 patients, or approximately 5%) of our original study population, and the impact of this exclusion is likely to be minimal. We did not utilize a washout period after the implementation of rMRI protocols in our institution. However, given the weekly trends observed in rMRI patterns, this would only lead to an underestimation of rMRI use (and an underestimation of CT reduction) since we included data from implementation without accounting for any washout period. We were unable to assess the test characteristics, including the rates of undetected pathology for the entire cohort, as not all patients received the reference standard (full MRI). Additionally, given the small number of patients available for this analysis, the confidence intervals surrounding these point estimates are wide and warrant further confirmation with larger sample sizes. Finally, we were unable to provide data with respect to use of anxiolytic medications, which were not available in our dataset.

## Conclusion

Widespread implementation of distinct rMRI protocols in our ED was associated with a 20% reduction in HCT, without missed diagnoses or increase in follow-up imaging. Important considerations with rMRI use compared to HCT in the ED setting include longer time to neuroimaging and LOS, and a higher rates of unsuccessful imaging. Despite these limitations, rMRI has the potential to supplant HCT for nontraumatic indications in the pediatric ED.

## Supplementary information

**Additional file 1: Figure S1.** a) number and b) percent of neuroimaging studies ordered as rMRI for each week of the rMRI study period.

**Additional file 2: Table S1.** Index neuroimaging findings by time period and imaging modality.

**Additional file 3: Table S2.** Diagnostic accuracy of index HCT and rMRI in 122 patients with follow-up full MRI within 14 days during the rMRI period.

**Additional file 4: Table S3.** Rates of neuroimaging across both time periods among all emergency department encounters for nontraumatic complaints.

**Additional file 5: Table S4.** Rates of neuroimaging across both time periods when performed as a sensitivity analysis (further exclusion of patients < 12 months with trauma).

**Additional file 6: Table S5.** Results of exploratory multivariable logistic regression identifying predictors associated with dichotomous outcome (CT versus no CT).

## Data Availability

The datasets generated and/or analysed during the current study are not publicly available due to privacy limitations but are available from the corresponding author on reasonable request and with the presence of a data use agreement.

## Abbreviations

ED: Emergency department
HCT: Head computerized tomography
IQR: Interquartile range
LOS: Length of stay
MRI: Magnetic resonance imaging
rMRI: Rapid magnetic resonance imaging
