# Supplementary figures and images for: Rapid brain MRI protocols reduce head computerized tomography use in the pediatric emergency department
Source: BMC Pediatr. 2020 Jan 13;20:14. doi: 10.1186/s12887-020-1919-3 (PMC6956479; doi:10.1186/s12887-020-1919-3)

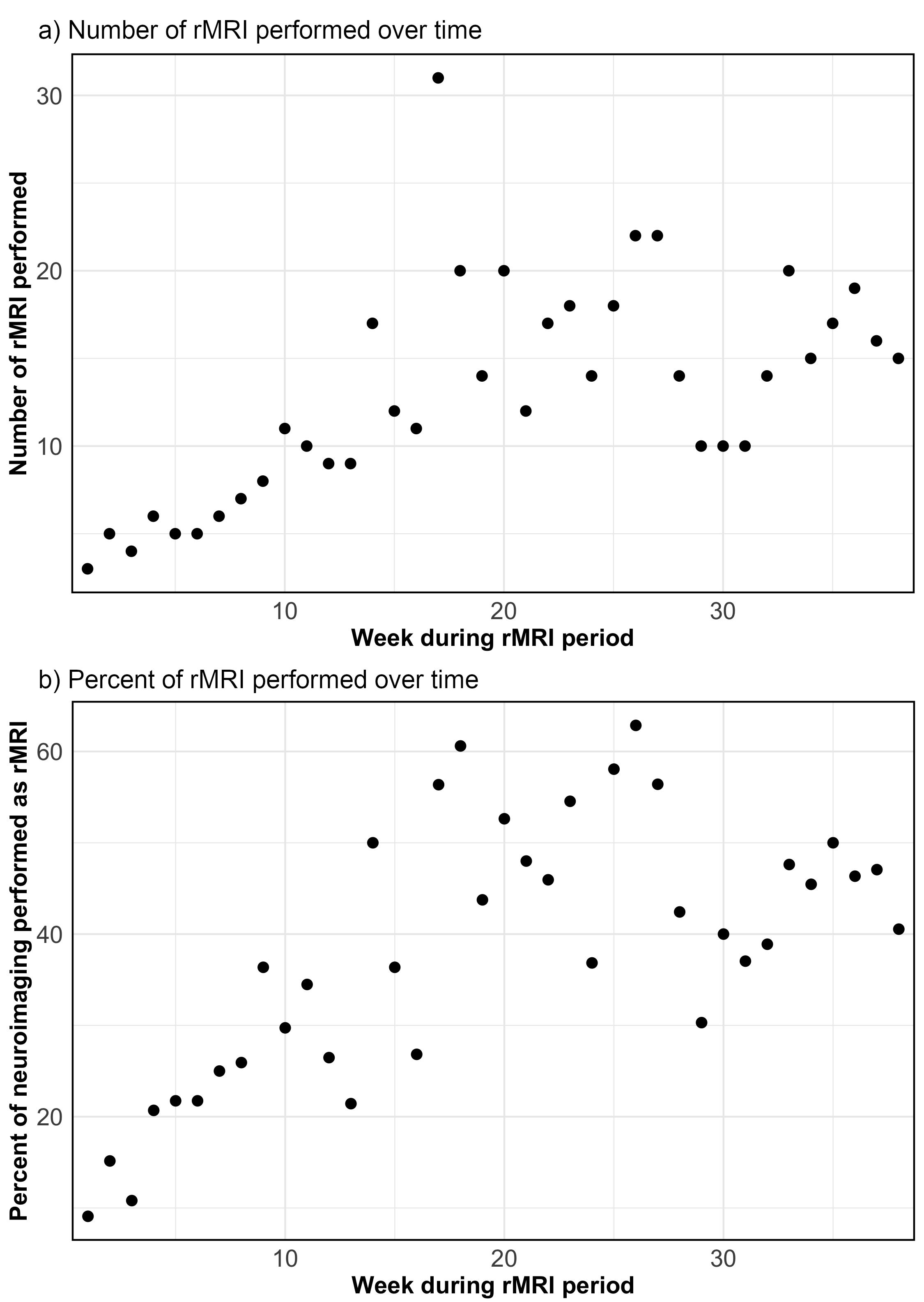

Supplement: Supplementary file 1 — Additional file 1: Figure S1. a) number and b) percent of neuroimaging studies ordered as rMRI for each week of the rMRI study period. [file 12887_2020_1919_MOESM1_ESM.tiff]
