# Supplementary material for: Rapid brain MRI protocols reduce head computerized tomography use in the pediatric emergency department
Source: BMC Pediatr. 2020 Jan 13;20:14. doi: 10.1186/s12887-020-1919-3 (PMC6956479; doi:10.1186/s12887-020-1919-3)
Supplement: Supplementary file 2 — Additional file 2: Table S1. Index neuroimaging findings by time period and imaging modality. [file 12887_2020_1919_MOESM2_ESM.docx]

**Supplementary Table 1.** Index neuroimaging findings by time period and imaging modality.

| **Variable** | **Interpretation** | **Control period**  **n (%)** | **rMRI period**  **n (%)** |
| --- | --- | --- | --- |
| **rMRI** | Positive | 11 (9.6) | 30 (6.0) |
|  | Negative | 93 (81.6) | 444 (88.1) |
|  | Unknown | 4 (3.5) | 14 (2.8) |
|  | Unsuccessful | 6 (5.3) | 16 (3.2) |
| **Head CT** | Positive | 139 (18.9) | 91 (14.4) |
|  | Negative | 586 (79.6) | 530 (83.6) |
|  | Unknown | 11 (1.5) | 13 (2.1) |
|  | Unsuccessful | 0 (0.0) | 0 (0.0) |
| **Full MRI** | Positive | 21 (10.4) | 28 (16.5) |
|  | Negative | 159 (78.7) | 120 (70.6) |
|  | Unknown | 17 (8.4) | 16 (9.4) |
|  | Unsuccessful | 5 (2.5) | 6 (3.5) |

rMRI rapid MRI; CT, computerized tomography; MRI, magnetic resonance imaging
