# Supplementary material for: Rapid brain MRI protocols reduce head computerized tomography use in the pediatric emergency department
Source: BMC Pediatr. 2020 Jan 13;20:14. doi: 10.1186/s12887-020-1919-3 (PMC6956479; doi:10.1186/s12887-020-1919-3)
Supplement: Supplementary file 3 — Additional file 3: Table S2. Diagnostic accuracy of index HCT and rMRI in 122 patients with follow-up full MRI within 14 days during the rMRI period. [file 12887_2020_1919_MOESM3_ESM.docx]

**Supplementary Table 2.** Diagnostic accuracy of index HCT and rMRI in 122 patients with follow-up full MRI within 14 days during the rMRI period

| **Radiologist** | **Index scan** | **Sensitivity % (95%CI)** | **Specificity**  **% (95%CI)** | **PPV**  **% (95%CI)** | **NPV**  **% (95%CI)** | **LR (+)**  **% (95%CI)** | **LR (-)**  **% (95%CI)** |
| --- | --- | --- | --- | --- | --- | --- | --- |
| Radiologist #1 | rMRI | 1.00 (0.69, 1.00) | 1.00 (0.87, 1.00) | 1.00 (0.69, 1.00) | 1.00 (0.87, 1.00) | n/a | n/a |
|  | Head CT | 0.82 (0.60, 0.95) | 0.95 (0.86, 0.99) | 0.86 (0.64, 0.97) | 0.93 (0.84, 0.98) | 16.36 (5.34, 50.17) | 0.19 (0.08, 0.47) |
| Radiologist #2 | rMRI | 1.00 (0.74, 1.00) | 1.00 (0.86, 1.00) | 1.00 (0.74, 1.00) | 1.00 (0.86, 1.00) | n/a | n/a |
|  | Head CT | 0.75 (0.55, 0.89) | 0.94 (0.85, 0.99) | 0.88 (0.68, 0.97) | 0.88 (0.77, 0.95) | 13.50 (4.40, 41.39) | 0.26 (0.14, 0.50) |

Note: Likelihood ratios could not be derived for rMRI because of the absence of false positives and false negatives among these patients. CT, computerized tomography; MRI, magnetic resonance imaging; rMRI, rapid MRI; PPV, positive predictive value; NPV, negative predictive value; LR(+), positive likelihood ratio, LR(-), negative likelihood ratio
