# Supplementary material for: Rapid brain MRI protocols reduce head computerized tomography use in the pediatric emergency department
Source: BMC Pediatr. 2020 Jan 13;20:14. doi: 10.1186/s12887-020-1919-3 (PMC6956479; doi:10.1186/s12887-020-1919-3)
Supplement: Supplementary file 4 — Additional file 4: Table S3. Rates of neuroimaging across both time periods among all emergency department encounters for nontraumatic complaints. [file 12887_2020_1919_MOESM4_ESM.docx]

**Supplementary Table 3.** Rates of neuroimaging across both time periods among all emergency department encounters for nontraumatic complaints.

| **Variable** | **Control period**  **(N=33,117)**  **n (%)** | **rMRI period (N=35,582)**  **n (%)** | **Difference in percent (95% CI)** |
| --- | --- | --- | --- |
| Any neuroimaging | 1,052 (3.2) | 1,308 (3.7) | 0.50 (0.23, 0.77) |
| rMRI | 114 (0.3) | 504 (1.4) | 1.1 (0.9, 1.2) |
| Head CT | 736 (2.2) | 634 (1.8) | -0.4 (-0.7, -0.2) |
| Full MRI | 202 (0.6) | 170 (0.5) | -0.1 (-0.2, 0.0) |
