# Supplementary material for: Rapid brain MRI protocols reduce head computerized tomography use in the pediatric emergency department
Source: BMC Pediatr. 2020 Jan 13;20:14. doi: 10.1186/s12887-020-1919-3 (PMC6956479; doi:10.1186/s12887-020-1919-3)
Supplement: Supplementary file 5 — Additional file 5: Table S4. Rates of neuroimaging across both time periods when performed as a sensitivity analysis (further exclusion of patients < 12 months with trauma). [file 12887_2020_1919_MOESM5_ESM.docx]

**Supplementary Table 4.** Rates of neuroimaging across both time periods when performed as a sensitivity analysis (further exclusion of patients <12 months with trauma)

| **Variable** | **Control period (N=810)**  **n (%)** | **rMRI period (N=1,009)**  **n (%)** | **Difference in percent (95% CI)** |
| --- | --- | --- | --- |
| rMRI | 77 (9.5) | 438 (43.4) | 33.9 (30.1, 37.7) |
| Head CT | 540 (66.7) | 410 (40.6) | -26.0 (-30.6, -21.5) |
| Full MRI | 193 (23.8) | 161 (16.0) | -7.9 (-11.7, -4.1) |

CT, computerized tomography; MRI, magnetic resonance imaging; rMRI rapid MRI; CI, confidence interval
