# Supplementary material for: Rapid brain MRI protocols reduce head computerized tomography use in the pediatric emergency department
Source: BMC Pediatr. 2020 Jan 13;20:14. doi: 10.1186/s12887-020-1919-3 (PMC6956479; doi:10.1186/s12887-020-1919-3)
Supplement: Supplementary file 6 — Additional file 6: Table S5. Results of exploratory multivariable logistic regression identifying predictors associated with dichotomous outcome (CT versus no CT). [file 12887_2020_1919_MOESM6_ESM.docx]

**Supplementary Table 5.** Results of exploratory multivariable logistic regression identifying predictors associated with dichotomous outcome (CT versus no CT).

| **Variable** | **Adjusted odds ratio**  **(95% confidence interval)** | **P value** |
| --- | --- | --- |
| ESI score |  |  |
| 1 or 2 | Ref | -- |
| 3 | 0.40 (0.31-0.53) | <0.01 |
| 4 or 5 | 0.64 (0.43-0.95) | 0.03 |
| Unknown | 1.05 (0.21-5.12) | 0.95 |
| Time period |  |  |
| Control period | Ref | -- |
| rMRI period | 0.42 (0.35-0.51) | <0.01 |
| Time of day |  |  |
| Day time | Ref | -- |
| Night time | 0.80 (0.67-0.95) | 0.02 |
| Age (years) | 0.95 (0.93-0.96) | <0.01 |
| Male sex | 1.39 (1.17-1.66) | <0.01 |

ESI, emergency severity index
